# High Pulse Wave Velocity Is Associated With Decreased Macular Vessel Density in Normal-Tension Glaucoma

**DOI:** 10.1167/iovs.62.10.12

**Published:** 2021-08-16

**Authors:** Taekjune Lee, Hyoung Won Bae, Gong Je Seong, Chan Yun Kim, Sang Yeop Lee

**Affiliations:** 1 Kim Eye Clinic, Cheongju-si, Republic of Korea; 2 Institute of Vision Research, Department of Ophthalmology, Severance Hospital, Yonsei University College of Medicine, Seoul, Republic of Korea; 3 Department of Ophthalmology, Yongin Severance Hospital, Yonsei University College of Medicine, Yongin-si, Republic of Korea

**Keywords:** pulse wave velocity, optical coherence tomography angiography, vessel density, normal-tension glaucoma

## Abstract

**Purpose:**

To investigate the relationship between pulse wave velocity (PWV) and retinal vessel density (VD) measured by optical coherence tomography angiography (OCTA) in patients with normal-tension glaucoma (NTG).

**Methods:**

This retrospective study included 103 patients with NTG and 109 healthy controls who underwent glaucoma examination and PWV measurements. Each group was classified into two subgroups according to a brachial-ankle PWV of 1400 cm/s. NTG was diagnosed when the maximum untreated intraocular pressure was < 21 mmHg on three repeated measurements obtained at different times in the presence of glaucomatous optic discs (neuroretinal rim thinning and excavation), peripapillary retinal nerve fiber layer defects, and glaucomatous visual field defects. Healthy controls did not have glaucomatous optic discs or visual field defects and exhibited normal retinal nerve fiber layer thickness. The interval between glaucoma examination and PWV measurements did not exceed six months. Univariate and multivariate logistic regression analyses were performed to identify factors associated with high PWV.

**Results:**

PWV was higher in the NTG group than in the control group, while peripapillary VD and macular VD (mVD) were lower (all *P* < 0.05). Stepwise logistic regression analysis revealed that high PWV was significantly associated with age, mean arterial pressure (MAP), and mVD in the NTG group. Meanwhile, high PWV was significantly associated with age, MAP, and low-density lipoprotein cholesterol levels in healthy controls.

**Conclusions:**

High PWV is associated with decreased mVD in NTG patients, suggesting that systemic arterial stiffness might be involved in the pathogenesis of NTG.

Glaucoma is characterized by progressive optic neuropathy with characteristic loss of optic nerve fibers and retinal ganglion cells (RGCs).^1^ There are two principal theories for the pathogenesis of glaucoma—a mechanical theory and a vascular theory.^2^ According to the mechanical theory, intraocular pressure (IOP) causes mechanical stretching of the lamina cribrosa and damage to RGC axons. The vascular theory states that insufficient blood supply due to either increased IOP or other risk factors causes a reduction in ocular blood flow.^3^^–^^5^ The vascular theory is particularly relevant to normal-tension glaucoma (NTG) since glaucomatous optic neuropathy often progresses in spite of low IOP in NTG. Therefore, numerous studies have investigated the relationship between vascular dysregulation and NTG.

Arterial stiffness, which refers to a loss of arterial elasticity, reflects vascular aging. Pulse wave velocity (PWV) is among the methods for representing systemic arterial stiffness and is a useful marker for predicting future cardiovascular events.^6^^,^^7^ PWV has been associated with eye diseases such as diabetic retinopathy,^8^^,^^9^ retinal vein occlusion,^10^ and age-related macular degeneration.^11^ Although several studies have investigated the relationship between PWV and glaucoma, their results have been inconsistent.^12^^–^^17^ Therefore, the role of PWV in glaucoma remains controversial.

The recent introduction of optical coherence tomography angiography (OCTA) allows for the noninvasive measurement of retinal vessel density (VD) in the peripapillary and macular areas.^18^^,^^19^ Studies using OCTA have reported decreased peripapillary and macular VD in glaucomatous eyes compared to healthy eyes.^19^^–^^22^ OCTA has also shown promise for monitoring glaucoma progression.^20^^,^^23^^,^^24^ Therefore, OCTA may aid in identifying the pathophysiology of glaucoma as it relates to vascular theory.

Although both measurements may play an important role in determining vascular pathophysiology in NTG, no studies have evaluated the association between PWV and OCTA parameters in NTG. Therefore, the present study aimed to investigate the relationship between PWV and OCTA parameters and to determine which ocular parameters are associated with PWV in patients with NTG.

## Methods

This retrospective cross-sectional study was performed in accordance with the tenets outlined in the Declaration of Helsinki and approved by the Institutional Review Board of Yonsei University (4-2020-0592). The requirement for informed consent was waived due to the retrospective nature of the study. We reviewed the medical records of all patients treated at Severance Hospital from January 2017 to July 2020.

A total of 109 healthy controls and 103 patients with NTG were included in this study. All participants underwent ophthalmologic examinations. All the examinations were carried out as usual for glaucoma patients, or patients who visited our hospital suspected of glaucoma. These included slit-lamp biomicroscopy, Goldmann applanation tonometry, gonioscopy, dilated fundus examination, measurement of best-corrected visual acuity (BCVA), and measurement of axial length (AXL) (IOL Master; Carl Zeiss Meditec, Dublin, CA, USA). Spectral-domain optical coherence tomography (OCT) (Cirrus HD-OCT, software v11.0; Carl Zeiss Meditec) and standard automated perimetry (Humphrey Field Analyzer II; Carl Zeiss Meditec) were performed to evaluate glaucomatous changes. PWV was usually measured at health check-ups, and blood tests included hemoglobin A1c (HbA1c), total cholesterol, triglycerides, high-density lipoprotein (HDL) cholesterol, low-density lipoprotein (LDL) cholesterol, estimated glomerular filtration rate (eGFR), and uric acid. The interval between glaucoma examination and PWV measurements did not exceed six months.

Inclusion criteria were as follows: (1) age ≥ 40 years; (2) able to confirm open angle on medical records; (3) BCVA ≥ 20/40; and (4) refractive error between +3 and –6 diopters (D) spherical and ±3 D cylindrical. NTG was diagnosed when the maximum untreated IOP was < 21 mm Hg on three repeated measurements obtained at different times on separate follow-up visits and in the presence of glaucomatous optic discs (neuroretinal rim thinning and excavation), peripapillary retinal nerve fiber layer (RNFL) defect, and glaucomatous visual field (VF) defects. Glaucomatous VF defects were defined if a cluster of at least three contiguous points had *P* < 0.05 on the pattern standard deviation plot where at least one of these points had *P* < 0.01, or if glaucoma hemifield test was outside normal limits.^25^ Glaucoma severity stage was based on the Hodapp-Parrish-Anderson criteria.^26^ The medical records were re-evaluated by two glaucoma specialists (T.L. and H.Y.B.). If the two specialists could not agree on a diagnosis of NTG, the final decision was made by a third glaucoma specialist (S.Y.L.). Healthy controls did not have glaucomatous optic discs or VF defects and exhibited normal RNFL thickness, which was within 95% of the internally set database for OCT results.

Patients with poor data quality or other ocular comorbidities were excluded: (1) signal strength less than 7, images with artifacts; (2) eyes with a diagnosis of pigment dispersion glaucoma, pseudoexfoliative glaucoma, or primary angle-closure glaucoma; (3) retinal disorder (diabetic retinopathy, age-related macular degeneration, retinal detachment, etc.); (4) history of ocular trauma or intraocular surgery; (5) history of brain disorder (brain hemorrhage, brain infarction, brain aneurysm, brain tumor, etc.); and (6) history of optic nerve disorder (optic neuritis, ischemic optic neuropathy, optic coloboma, etc.).

For patients with NTG, the eye with a more severe glaucoma status was chosen as the study eye. If patients had a similar glaucoma severity in both eyes, the study eye was randomly selected. For patients with healthy eyes, the study eye was also randomly selected.

### Pulse Wave Velocity

Brachial-ankle PWV (baPWV) was measured using volume-plethysmography (VP-1000 plus; Omron HealthCare Co. Ltd., Kyoto, Japan). Trained technicians and physicians placed the pressure cuffs on both arms and both ankles of the patient and performed the measurement after the patient had rested for approximately 10 minutes in the supine position. The travel path from body height was extrapolated, and baPWV was computed automatically by dividing the time difference between the pulse waves that were transmitted to the brachial and ankle arteries by the travel path. The faster value of the right- and left-side baPWV was used for analysis. The device also measured blood pressure (BP) at the same time. Systolic BP (SBP) and diastolic BP (DBP) were measured, and mean arterial pressure (MAP) was calculated as follows: [SBP + (2 × DBP)]/3.

We set a baPWV of 1400 cm/s as the reference value since this is what has been identified as an independent risk factor in the Framingham score and can distinguish patients with atherosclerotic cardiovascular disease.^27^^,^^28^ It is also adopted as a reference value in the 2017 American College of Cardiology/American Heart Association (ACC/AHA) Guideline for the Prevention, Detection, Evaluation, and Management of High Blood Pressure in Adults.^29^

### OCTA

Under pupil dilation, OCTA (AngioPlex; Carl Zeiss Meditec) images of the peripapillary and macular areas were obtained using a 6 × 6 mm scan. The optical microangiography algorithm analyzed the changes in the complex signal (both intensity and phase changes contained within sequential B-scans performed at the same position) and then generated en face microvascular images. In the 6 × 6 mm scan, there were 350 A-scans in each B-scan along the horizontal dimension, and 350 B-scans were repeated twice at each location. The boundaries of the superficial and deep retinal layers were determined automatically. The inner surface of the superficial retinal layer (SRL) was defined by the internal limiting membrane. The outer surface of the SRL was denoted by the outer border of inner plexiform layer. The segmentation software automatically detected the boundaries of the retinal layers from the structural OCT cross-sectional images by measuring the gradient of OCT signals to create SRL en face images. With the aid of innate software for AngioPlex OCTA, VD was defined as the total length of perfused vasculature per unit area in a region of measurement. The innate software automatically calculated the VD in the center circle with a diameter of 6 mm. It included all the vessels in the area, including large vessels. Thus, peripapillary VD (pVD) and macular VD (mVD) were calculated. All examinations were individually reviewed by two glaucoma specialists (T.L. and H.W.B.), and images with artifacts or signal strength less than 7 were excluded.

### Statistical Analysis

All statistical analyses were performed using the commercially available software SPSS (ver. 23.0; SPSS Inc., Chicago, IL, USA). Continuous variables were summarized as mean and standard deviation. Categorical variables were summarized as frequencies and percentages. Demographic and clinical data between groups were compared using independent *t*-tests for continuous parameters and chi-square tests for categorical parameters. Univariate logistic regression analysis was used to identify the factors associated with high PWV in each group. Variables with *P* values less than 0.2 during the univariate step were further included in a multivariate backward stepwise logistic regression model. In all analyses, *P* values less than 0.05 were considered to indicate statistical significance.

## Results

In total, 109 eyes of 109 healthy controls and 103 eyes of 103 patients with NTG were enrolled. Table 1 presents the baseline characteristics of the study population. Patients in the NTG group were significantly older (*P* = 0.006) and more frequently had hypertension (*P* = 0.014) than healthy controls. SBP and MAP were significantly higher (*P* = 0.011, *P* = 0.039, respectively), while total cholesterol and LDL cholesterol were lower (*P* = 0.030, *P* = 0.004, respectively) in the NTG group than in the control group. PWV was also much faster in the NTG group than in the control group (*P* = 0.026).

**Table 1.** Demographic Characteristics in the Healthy Control and NTG Groups

	Healthy (*n* = 109)	NTG (*n* = 103)	*P* Value
Systemic parameters			
Age (years)	58.1 ± 9.1	61.6 ± 9.3	0.006
Male (%)	57 (52.3)	55 (53.4)	0.872
Body mass index (kg/m^2^)	24.0 ± 3.5	24.0 ± 3.0	0.210
Pulse wave velocity (cm/s)	1436.6 ± 275.7	1537.8 ± 369.0	0.025
Hypertension (%)	31 (28.4)	46 (44.7)	0.014
Systolic BP (mmHg)	122.2 ± 15.3	127.9 ± 17.1	0.011
Diastolic BP (mmHg)	75.1 ± 10.7	77.3 ± 10.2	0.131
Mean arterial pressure (mmHg)	90.8 ± 11.8	94.2 ± 11.8	0.039
Diabetes mellitus (%)	22 (20.2)	22 (21.4)	0.833
HbA1c (%)	5.9 ± 1.2	6.0 ± 1.2	0.550
Dyslipidemia (%)	33 (30.3)	37 (35.9)	0.382
Total cholesterol (mg/dL)	189.2 ± 40.5	176.6 ± 43.7	0.030
Triglycerides (mg/dL)	103.4 ± 68.7	127.5 ± 84.2	0.024
HDL cholesterol (mg/dL)	53.9 ± 12.8	50.6 ± 11.8	0.055
LDL cholesterol (mg/dL)	116.3 ± 38.3	101.2 ± 38.1	0.004
Chronic kidney disease (%)	3 (2.8)	9 (8.7)	0.059
eGFR (ml/min)	92.2 ± 14.0	86.9 ± 18.5	0.019
Uric acid	5.3 ± 1.4	5.5 ± 1.6	0.363
Ocular parameters			
Intraocular pressure (mmHg)	14.0 ± 2.9	13.3 ± 2.0	0.041
Axial length (mm)	24.2 ± 1.2	24.3 ± 1.4	0.340
Spherical equivalent (D)	−0.6 ± 1.9	−1.4 ± 2.8	0.022
Average RNFL thickness (µm)	94.1 ± 8.9	77.4 ± 12.3	<0.001
Average GCIPL thickness (µm)	82.0 ± 6.0	71.8 ± 8.3	<0.001
Peripapillary VD (mm^−1^)	17.2 ± 1.0	16.2 ± 1.6	<0.001
Macular VD (mm^−1^)	16.7 ± 1.7	15.9 ± 1.9	0.002
Visual field mean deviation (dB)		−3.7 ± 3.6	
Glaucoma severity			
Early		86 (83.5%)	
Moderate to severe		17 (16.5%)	

NTG: normal-tension glaucoma; BP: blood pressure; HbA1c: hemoglobin A1c; HDL: high-density lipoprotein; LDL: low-density lipoprotein; eGFR: estimated glomerular filtration rate; RNFL: retinal nerve fiber layer; GCIPL: ganglion cell–inner plexiform layer; VD: vessel density.

**Figure 1.**
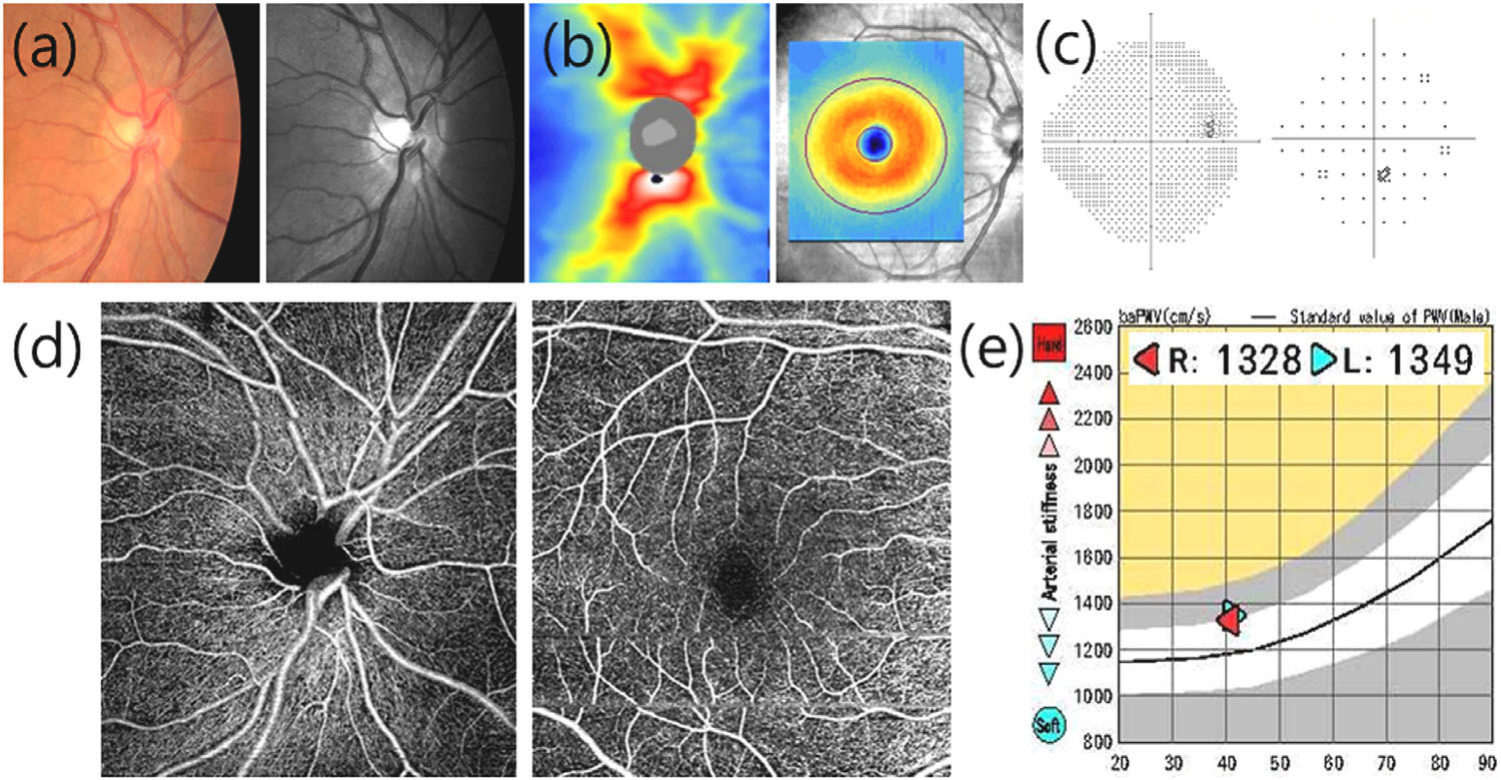
A representative case in the healthy control group. All examination results were normal. (**a**) Color and red-free fundus photography; (**b**) RNFL and GCIPL thickness on OCT; (**c**) VF; (**d**) Peripapillary VD and macular VD on OCTA; (**e**) baPWV. RNFL: retinal nerve fiber layer; GCIPL: ganglion cell–inner plexiform layer; OCT: optical coherence tomography; VF: visual field; VD: vessel density; OCTA: optical coherence tomography angiography; baPWV: brachial-ankle pulse wave velocity.

**Figure 2.**
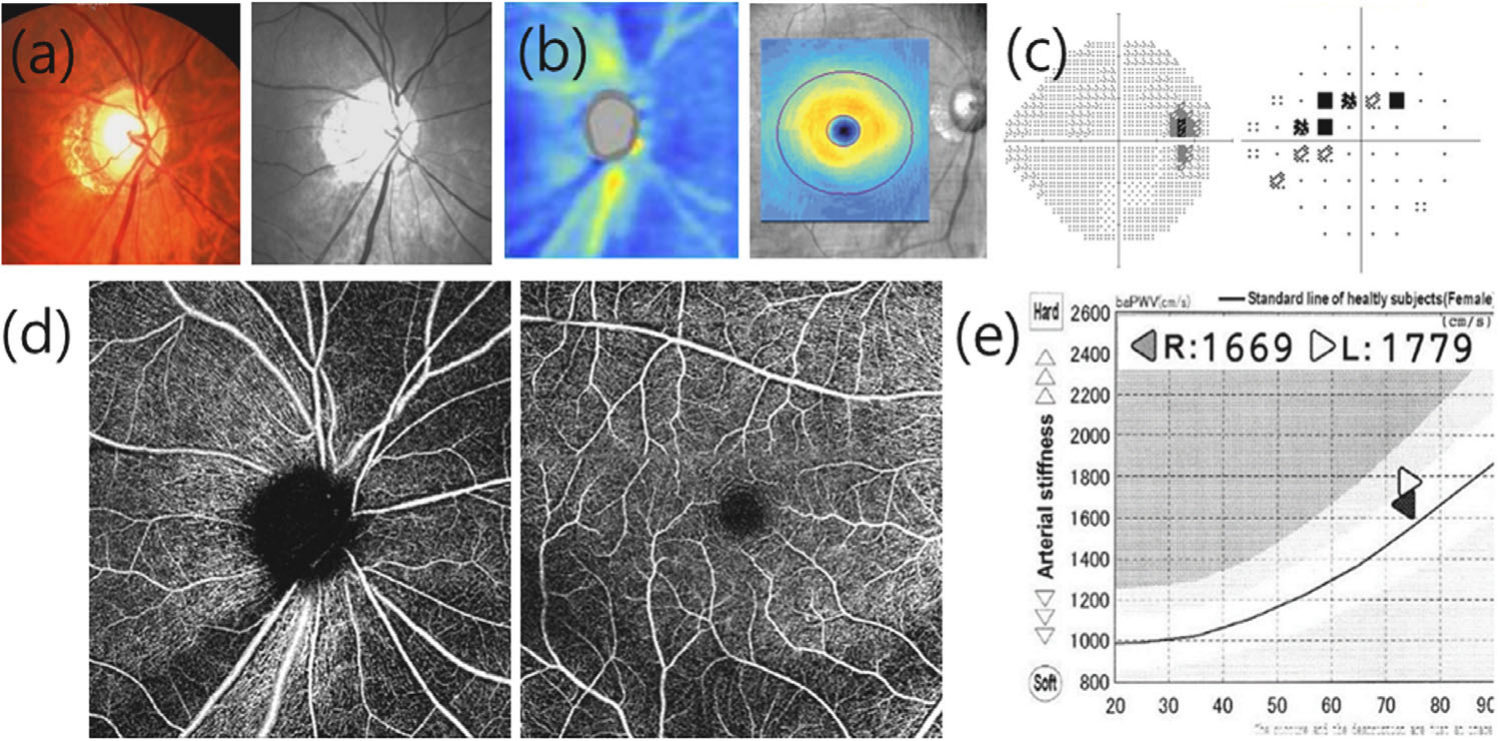
A representative case in the NTG group. (**a**) Color and red-free fundus photography; (**b**) OCT showed RNFL and GCIPL defects in the inferior region; (**c**) Paracentral VF defect in the superior hemifield; (**d**) Peripapillary and macular VD were reduced on OCTA; (**e**) baPWV was increased to 1669 cm/s (*right*) and 1779 cm/s (*left*). RNFL: retinal nerve fiber layer; GCIPL: ganglion cell–inner plexiform layer; OCT: optical coherence tomography; VF: visual field; VD: vessel density; OCTA: optical coherence tomography angiography; baPWV: brachial-ankle pulse wave velocity.

Patients in the NTG group exhibited reduced pVD and mVD when compared to healthy controls (*P* < 0.001, *P* = 0.002, respectively). Average RNFL thickness and average ganglion cell–inner plexiform layer (GCIPL) thickness were thinner in the NTG group than in the control group (all *P* < 0.001). IOP was slightly lower (*P* = 0.041), and spherical equivalents (SE) were more myopic (*P* = 0.022) in the NTG group than in the control group, although the difference was relatively small. In the NTG group, the majority of patients (86 eyes, 83.5%) were in the early stage of glaucoma, with a mean deviation better than –6 dB in the VF test. Figure 1 and Figure 2 depict representative cases in the healthy control and NTG groups, respectively.

Table 2 shows demographic characteristics for the low PWV group (PWV < 1400 cm/s) and high PWV group (PWV ≥ 1400 cm/s) in healthy controls. The high PWV group was significantly older (*P* < 0.001), more hyperopic (*P* = 0.008), and more frequently had hypertension (HTN) (*P* < 0.001) than the low PWV group.

**Table 2.** Demographic Characteristics of the Low PWV and High PWV Groups in Healthy Controls

	Low PWV (*n* = 44)	High PWV (*n* = 59)	
	(PWV < 1400 cm/s)	(PWV ≥ 1400 cm/s)	*P* Value
Systemic parameters			
Age (years)	54.6 ± 8.0	62.5 ± 8.4	<0.001
Male (%)	34	23	0.417
Body mass index (kg/m^2^)	24.0 ± 3.8	24.0 ± 3.2	0.998
Pulse wave velocity (cm/s)	1247.6 ± 129.6	1676.7 ± 219.1	<0.001
Hypertension (%)	9	22	<0.001
Systolic BP (mmHg)	115.9 ± 12.4	130.3 ± 15.1	<0.001
Diastolic BP (mmHg)	71.7 ± 9.4	79.5 ± 10.9	<0.001
Mean arterial pressure (mmHg)	86.4 ± 9.9	96.4 ± 11.8	<0.001
Diabetes (%)	9	13	0.111
HbA1c (%)	5.7 ± 1.1	6.1 ± 1.2	0.087
Dyslipidemia (%)	17	16	0.538
Total cholesterol (mg/dL)	194.6 ± 44.2	182.4 ± 34.7	0.121
Triglycerides (mg/dL)	97.4 ± 52.5	110.9 ± 84.7	0.312
HDL cholesterol (mg/dL)	54.4 ± 12.5	53.3 ± 13.2	0.660
LDL cholesterol (mg/dL)	122.1 ± 39.7	109.1 ± 35.6	0.080
Chronic kidney disease (%)	2	1	0.705
eGFR (ml/min)	94.0 ± 14.3	90.0 ± 13.4	0.142
Uric acid (mg/dL)	5.3 ± 1.4	5.4 ± 1.3	0.785
Ocular parameters			
Intraocular pressure (mmHg)	13.9 ± 2.9	14.1 ± 3.0	0.771
Axial length (mm)	24.3 ± 1.2	23.9 ± 1.1	0.065
Spherical equivalent (diopters)	−1.1 ± 1.9	−0.1 ± 1.7	0.008
Average RNFL thickness (µm)	93.9 ± 8.2	94.5 ± 9.7	0.698
Average GCIPL thickness (µm)	82.3 ± 5.6	81.6 ± 6.5	0.574
Peripapillary VD (mm^−1^)	17.3 ± 1.1	17.1 ± 0.7	0.212
Macular VD (mm^−1^)	16.9 ± 1.7	16.5 ± 1.6	0.157

PWV: pulse wave velocity; BP: blood pressure; HbA1c: hemoglobin A1c; HDL: high-density lipoprotein; LDL: low-density lipoprotein; eGFR: estimated glomerular filtration rate; RNFL: retinal nerve fiber layer; GCIPL: ganglion cell–inner plexiform layer; VD: vessel density.

A univariate logistic regression analysis revealed that high PWV was significantly associated with age, SBP, DBP, MAP, and SE (all *P* < 0.05). Variables with *P* values less than 0.2 were further enrolled in a multivariate model. Since SBP, DBP, and MAP have multicollinearity, MAP was chosen for the multivariate model. A multivariate backward stepwise logistic regression analysis revealed that age (odds ratio (OR) = 1.151, 95% confidence interval (CI) = 1.072-1.237, *P* < 0.001), MAP (OR = 1.117, 95% CI = 1.060–1.178), and LDL cholesterol (OR = 0.987, 95% CI = 0.974–1.000, *P* = 0.046) were significantly associated with high PWV in healthy controls (Table 3).

**Table 3.** Univariate and Multivariate Backward Stepwise Logistic Regression Analysis for High PWV (PWV ≥ 1400 cm/s) in the Healthy Control Group

	Univariate			Multivariate		
	Odds Ratio	95% CI	*P* Value	Odds Ratio	95% CI	*P* Value
Age, years	1.126	1.064–1.193	<0.001	1.151	1.072–1.237	<0.001
Sex, male	1.369	0.641–2.924	0.418			
Body mass index (kg/m^2^)	1.000	0.897–1.114	0.998			
Systolic BP (mmHg)	1.083	1.044–1.123	<0.001			
Diastolic BP (mmHg)	1.086	1.036–1.137	0.001			
Mean arterial pressure (mmHg)	1.098	1.048–1.151	<0.001	1.117	1.060–1.178	<0.001
HbA1c (%)	1.358	0.936–1.971	0.107			
Total cholesterol (mg/dL)	0.992	0.983–1.002	0.123			
Triglycerides (mg/dL)	1.003	0.997–1.009	0.317			
HDL cholesterol (mg/dL)	0.993	0.964–1.023	0.657			
LDL cholesterol (mg/dL)	0.991	0.981–1.001	0.083	0.987	0.974–1.000	0.046
eGFR (ml/min)	0.979	0.952–1.007	0.146			
Uric acid (mg/dL)	1.040	0.787–1.376	0.782			
Intraocular pressure (mmHg)	1.020	0.895–1.161	0.768			
Axial length (mm)	0.728	0.517–1.025	0.069			
Spherical equivalent (diopters)	1.358	1.072–1.721	0.011			
Average RNFL thickness (µm)	1.009	0.966–1.053	0.695			
Average GCIPL thickness (µm)	0.982	0.921–1.046	0.570			
Peripapillary VD (mm^−1^)	0.782	0.522–1.171	0.232			
Macular VD (mm^−1^)	0.850	0.678–1.065	0.158			
Hosmer-Lemeshow *P* value: 0.068						
Nagelkerke R^2^ = 0.492						

Variables with *P* values less than 0.2 during the univariate step were further enrolled in a backward stepwise logistic regression model.

PWV: pulse wave velocity; CI: confidence interval; BP: blood pressure; Hb: hemoglobin; HDL: high-density lipoprotein; LDL: low-density lipoprotein; eGFR: estimated glomerular filtration rate; RNFL: retinal nerve fiber layer; GCIPL: ganglion cell–inner plexiform layer; VD: vessel density.

Table 4 shows demographic characteristics for the low PWV group and high PWV group in patients with NTG. The high PWV group was significantly older (*P* < 0.001) and more frequently had HTN (*P* = 0.002), diabetes mellitus (DM) (*P* = 0.009), and chronic kidney disease (*P* = 0.007) than the low PWV group. SBP, DBP, MAP (all *P* < 0.001), and HbA1c (*P* = 0.022) were significantly higher, while total cholesterol (*P* = 0.030) and LDL cholesterol (*P* = 0.017) were lower in the high PWV group than in the low PWV group. In addition, the high PWV group had significantly shorter AXL (*P* = 0.008), more hyperopic SE (*P* = 0.010) and lower pVD (*P* = 0.036) and mVD (*P* < 0.001) than the low PWV group.

**Table 4.** Demographic Characteristics of the Low PWV and High PWV Groups in the NTG Group

	Low PWV (*n* = 44)	High PWV (*n* = 59)	
	(PWV < 1400 cm/s)	(PWV ≥ 1400 cm/s)	*P* Value
Systemic parameters			
Age (years)	56.3 ± 7.2	65.6 ± 8.7	<0.001
Male (%)	28	27	0.072
Body mass index (kg/m^2^)	24.3 ± 3.1	23.6 ± 2.8	0.232
Pulse wave velocity (cm/s)	1242.0 ± 90.4	1758.3 ± 342.8	<0.001
Hypertension (%)	12	34	0.002
Systolic BP (mmHg)	116.7 ± 11.6	136.3 ± 15.7	<0.001
Diastolic BP (mmHg)	72.6 ± 8.3	80.7 ± 10.2	<0.001
Mean arterial pressure (mmHg)	87.3 ± 9.0	99.3 ± 11.0	<0.001
Diabetes (%)	4	18	0.009
HbA1c (%)	5.7 ± 0.8	6.2 ± 1.4	0.022
Dyslipidemia (%)	14	2	0.453
Total cholesterol (mg/dL)	187.4 ± 37.9	168.6 ± 46.2	0.030
Triglycerides (mg/dL)	129.2 ± 91.2	126.3 ± 79.4	0.860
HDL cholesterol (mg/dL)	51.9 ± 10.6	49.7 ± 12.7	0.356
LDL cholesterol (mg/dL)	111.5 ± 35.4	93.5 ± 38.5	0.017
Chronic kidney disease (%)	0	9	0.007
eGFR (ml/min)	94.7 ± 14.0	81.1 ± 19.4	<0.001
Uric acid	5.8 ± 1.6	5.3 ± 1.5	0.121
Ocular parameters			
Intraocular pressure (mmHg)	12.9 ± 2.0	13.6 ± 1.9	0.057
Axial length (mm)	24.8 ± 1.5	24.0 ± 1.3	0.008
Spherical equivalent (D)	−2.2 ± 3.0	−0.8 ± 2.5	0.010
Average RNFL thickness (µm)	78.7 ± 12.2	76.4 ± 12.4	0.359
Average GCIPL thickness (µm)	72.7 ± 8.1	71.1 ± 8.4	0.343
Peripapillary VD (mm^−1^)	16.6 ± 1.6	15.9 ± 1.5	0.036
Macular VD (mm^−1^)	16.8 ± 1.7	15.3 ± 1.8	<0.001
Visual field mean deviation (dB)	−4.0 ± 4.7	−3.4 ± 2.4	0.436

PWV: pulse wave velocity; NTG: normal-tension glaucoma; BP: blood pressure; HbA1c: hemoglobin A1c; HDL: high-density lipoprotein; LDL: low-density lipoprotein; eGFR: estimated glomerular filtration rate; RNFL: retinal nerve fiber layer; GCIPL: ganglion cell–inner plexiform layer; VD: vessel density.

A univariate logistic regression analysis revealed that high PWV was significantly associated with age, SBP, DBP, MAP, HbA1c, total cholesterol, LDL cholesterol, eGFR, AXL, SE, pVD, and mVD (all *P* < 0.05). A multivariate backward stepwise logistic regression analysis revealed that age (OR = 1.228, 95% CI = 1.113–1.356, *P* < 0.001), MAP (OR = 1.221, 95% CI = 1.112–1.341, *P* < 0.001), and mVD (OR = 0.655, 95% CI = 0.445–0.964, *P* = 0.032) were significantly associated with high PWV (Table 5).

**Table 5.** Univariate and Multivariate Backward Stepwise Logistic Regression Analysis for High PWV (PWV ≥ 1400 cm/s) in NTG Group

	Univariate			Multivariate		
	Odds Ratio	95% CI	*P* Value	Odds Ratio	95% CI	*P* Value
Age, years	1.153	1.083–1.229	<0.001	1.228	1.113–1.356	<0.001
Sex, male	2.074	0.932–4.615	0.074			
Body mass index (kg/m^2^)	0.921	0.805–1.054	0.233			
Systolic BP (mmHg)	1.123	1.070–1.178	<0.001			
Diastolic BP (mmHg)	1.099	1.045–1.155	<0.001			
Mean arterial pressure (mmHg)	1.131	1.071–1.194	<0.001	1.221	1.112–1.341	<0.001
HbA1c (%)	1.611	1.003–2.589	0.049			
Total cholesterol (mg/dL)	0.990	0.980–0.999	0.034			
Triglycerides (mg/dL)	1.000	0.995–1.004	0.859			
HDL cholesterol (mg/dL)	0.984	0.952–1.018	0.353			
LDL cholesterol (mg/dL)	0.987	0.976–0.998	0.020			
eGFR (ml/min)	0.948	0.919–0.978	0.001			
Uric acid (mg/dL)	0.815	0.628–1.058	0.124			
Intraocular pressure (mmHg)	1.220	0.991-1.503	0.061			
Axial length (mm)	0.675	0.498–0.915	0.011			
Spherical equivalent (diopters)	1.214	1.041–1.417	0.014			
Average RNFL thickness (µm)	0.985	0.954–1.017	0.356			
Average GCIPL thickness (µm)	0.977	0.931–1.025	0.341			
Peripapillary VD (mm^−1^)	0.750	0.570–0.988	0.041			
Macular VD (mm^−1^)	0.605	0.461–0.796	<0.001	0.655	0.445–0.964	0.032
Hosmer-Lemeshow *P* value: 0.595						
Nagelkerke R^2^ = 0.672						

Variables with *P* values less than 0.2 during the univariate step were further enrolled in a backward stepwise logistic regression model.

PWV: pulse wave velocity; NTG: normal-tension glaucoma; BP: blood pressure; HbA1c: hemoglobin A1c; HDL: high-density lipoprotein; LDL: low-density lipoprotein; eGFR: estimated glomerular filtration rate; RNFL: retinal nerve fiber layer; GCIPL: ganglion cell–inner plexiform layer; VD: vessel density.

## Discussion

To our knowledge, this is the first study to evaluate the relationship between PWV and retinal VD in NTG. Our findings confirmed that high PWV is associated with decreased mVD in patients with NTG, indicating that systemic arterial stiffness may play a role in the vascular pathophysiology of NTG. The vascular theory is thought to be a main factor influencing NTG,^30^^,^^31^ given its relevance to systemic vascular dysregulation, including vascular and endothelial diseases,^32^ vasospasm syndrome,^33^ and migraine.^34^^,^^35^ PWV reflects systemic arterial stiffness, and OCTA evaluates the microvascular status of the eye, which is an end organ. The relevance of these two parameters supports the vascular theory of NTG and suggests the importance of systemic vessel status evaluations in patients with NTG.

Although several studies have investigated the relationship between PWV and glaucoma, they have yielded conflicting results. The Rotterdam Eye Study^15^ first showed that participants with an increased carotid-femoral PWV (cfPWV) and especially those with a low carotid distensibility coefficient, both indicative of high arterial stiffness, had a higher prevalence of primary open-angle glaucoma (POAG), although the results were not statistically significant. In other studies regarding the relationship between cfPWV and glaucoma, cfPWV exhibited a stepwise increase from healthy controls to patients with POAG and those with pseudoexfoliative glaucoma,^13^ and such increases in cfPWV were associated with pseudoexfoliative glaucoma.^17^ Other studies evaluated the relationship between baPWV and glaucoma. One study reported that baPWV was positively associated with glaucoma and NTG in particular, while other studies failed to identify a significant difference in baPWV between patients with NTG and healthy controls.^12^^,^^14^ These diverse results may be due to the different glaucoma subgroups and different modalities used to measure PWV.

The two most frequently applied measurements of PWV are cfPWV and baPWV. We used baPWV due to its simplicity and noninvasiveness. baPWV is measured simply by wrapping pressure cuffs around the four extremities without taking off clothes and is an independent marker of future cardiovascular events.^6^^,^^7^ baPWV may be applicable even in those with a low risk of cardiovascular disease (CVD),^6^ while cfPWV is thought to be applicable in patients with a high risk of CVD.^36^ Therefore, baPWV measurements are included in routine health check-up programs in South Korea. Considering the relationship between these measurements and other ocular disorders such as diabetic retinopathy,^8^^,^^9^ retinal vein occlusion,^10^ and age-related macular degeneration,^11^ the result of our study—which identified a correlation between PWV and mVD measured by OCTA in patients with NTG—suggests the necessity of ophthalmologic examination for patients who have high PWV. Although there is a lack of evidence now, if further research confirms that PWV is not only related with glaucoma but also a risk factor that increases the risk of glaucoma development or progression, PWV will be an important test to evaluate the condition of glaucoma patients.

It is well known that NTG is associated with systemic vascular factors and impaired ocular blood flow.^37^ Therefore, a relationship between decreased VD and increased systemic arterial stiffness can be expected in patients with NTG. However, the mechanism underlying the association between high systemic arterial stiffness and decreased mVD in NTG remains unclear. One possible explanation is that, although baPWV reflects the stiffness of medium- to large-sized arteries, excessive pressure pulsatility may affect microvascular remodeling and impair the regulation of local blood flow, which can lead to diffuse microscopic tissue damage.^38^ This notion is supported by another study, which demonstrated that increased aortic stiffness is inversely correlated with retinal arterial lumen diameter and increased microvascular resistance.^39^ Moreover, one study reported that untreated NTG eyes had stiffer retinal vessels and that vessel rigidity correlates with the level of glaucomatous damage.^40^ Further studies are required to identify the mechanism underlying the relationship between systemic arterial stiffness and retinal vessel damage.

In this study, baPWV was significantly associated with mVD but not with pVD. One possible explanation is that there is an NTG subtype in which glaucomatous changes first occur in the macular GCIPL rather than the peripapillary RNFL.^41^^,^^42^ Since the SRL of OCTA is composed of the area from the inner limiting membrane to the inner plexiform layer and our study group mainly consisted of patients with early-stage NTG, mVD may have been affected and impaired earlier. Further studies are required to determine the association between PWV and OCTA at different glaucoma stages and in different subtypes of glaucoma along with the pattern of VF progression.

Our findings also revealed that high PWV is associated with age and MAP in both groups. Age is a strong predictor of PWV,^43^ and PWV is well known to be dependent on blood pressure at the time of measurement.^44^^–^^46^ Age and BP are also among the important factors to consider when evaluating retinal VD.^47^^–^^49^ Therefore, age and BP should be considered when interpreting the relationship between PWV and mVD. Moreover, PWV has been associated with DM,^50^^,^^51^ abnormal lipid metabolism,^52^^,^^53^ smoking,^54^ elevated uric acid levels,^55^ high body mass index,^56^ and chronic kidney disease.^57^^,^^58^ Although we included all the possible systemic factors influencing PWV as covariates, mVD was still significantly associated with PWV in patients with NTG in the multivariate logistic regression analysis.

There are some limitations in this study. First, this was a retrospective cross-sectional study, and the association discovered here may not imply a causal relationship. Cautions are needed when interpreting the results. Further study is needed for the mechanism underlying the relationship between PWV and mVD in NTG. Second, we only included data with good image quality, as signal strength is an important factor when analyzing OCTA images. Thus, it is possible that selection bias occurred. However, these conditions would have contributed to increasing the reliability of our study results. Third, the sample in this study comprised mostly patients with early-stage NTG (86 eyes, 83.5%). This must be considered when using the results of our study to inform clinical practice, as the relationship may differ in patients with more advanced NTG or other types of glaucoma, such as POAG. Studies comparing POAG with NTG may aid in clarifying the significance of PWV in glaucoma. Lastly, because of its retrospective study design, we were unable to account for all the factors that affect the measurement of PWV and OCTA. Further studies that consider all possible factors are required.

In conclusion, our study demonstrates that high PWV is associated with decreased mVD in patients with NTG, indicating that systemic arterial stiffness might be involved in the pathogenesis of NTG. These results further indicate that clinical diagnosis and treatment of NTG may require consideration of systemic arterial stiffness, which can be confirmed by measuring PWV. In addition, further research regarding the role of systemic arterial stiffness in the pathogenesis of NTG is needed.

## References

[1] Weinreb RN, Khaw PT. Primary open-angle glaucoma. *Lancet*. 2004; 363: 1711–1720. 15158634 10.1016/S0140-6736(04)16257-0

[2] Fechtner RD, Weinreb RN. Mechanisms of optic nerve damage in primary open angle glaucoma. *Surv Ophthalmol*. 1994; 39: 23–42. 7974188 10.1016/s0039-6257(05)80042-6

[3] Cioffi GA, Sullivan P. The effect of chronic ischemia on the primate optic nerve. *Eur J Ophthalmol*. 1999; 9(Suppl 1): S34–S36. 10230604 10.1177/112067219900901S12

[4] Flammer J, Orgul S, Costa VP, et al. The impact of ocular blood flow in glaucoma. *Prog Retin Eye Res*. 2002; 21: 359–393. 12150988 10.1016/s1350-9462(02)00008-3

[5] Katai N, Yoshimura N. Apoptotic retinal neuronal death by ischemia-reperfusion is executed by two distinct caspase family proteases. *Invest Ophthalmol Vis Sci*. 1999; 40: 2697–2705. 10509668

[6] Ohkuma T, Ninomiya T, Tomiyama H, et al. Brachial-ankle pulse wave velocity and the risk prediction of cardiovascular disease: an individual participant data meta-analysis. *Hypertension*. 2017; 69: 1045–1052. 28438905 10.1161/HYPERTENSIONAHA.117.09097

[7] Tomiyama H, Matsumoto C, Shiina K, et al. Brachial-ankle PWV: current status and future directions as a useful marker in the management of cardiovascular disease and/or cardiovascular risk factors. *J Atheroscler Thromb*. 2016; 23: 128–146. 26558401 10.5551/jat.32979

[8] Ogawa O, Hiraoka K, Watanabe T, et al. Diabetic retinopathy is associated with pulse wave velocity, not with the augmentation index of pulse waveform. *Cardiovasc Diabetol*. 2008; 7: 11. 18439284 10.1186/1475-2840-7-11 PMC2377239

[9] Liu SC, Chuang SM, Shih HM, et al. High pulse wave velocity is associated with the severity of diabetic retinopathy in patients with type 2 diabetes. *J Investig Med*. 2020; 68: 1159–1165. 10.1136/jim-2019-001240 32595133

[10] Kaderli AA, Kaderli B, Gullulu S, et al. Impaired aortic stiffness and pulse wave velocity in patients with branch retinal vein occlusion. *Graefes Arch Clin Exp Ophthalmol*. 2010; 248: 369–374. 20084390 10.1007/s00417-009-1271-7

[11] Sato E, Feke GT, Appelbaum EY, et al. Association between systemic arterial stiffness and age-related macular degeneration. *Graefes Arch Clin Exp Ophthalmol*. 2006; 244: 963–971. 16411106 10.1007/s00417-005-0201-6

[12] Bossuyt J, Vandekerckhove G, De Backer TL, et al. Vascular dysregulation in normal-tension glaucoma is not affected by structure and function of the microcirculation or macrocirculation at rest: a case-control study. *Medicine (Baltimore)*. 2015; 94: e425. 25590850 10.1097/MD.0000000000000425 PMC4602537

[13] Bourouki E, Oikonomou E, Moschos M, et al. Pseudoexfoliative glaucoma, endothelial dysfunction, and arterial stiffness: the role of circulating apoptotic endothelial microparticles. *J Glaucoma*. 2019; 28: 749–755. 31188231 10.1097/IJG.0000000000001303

[14] Chiba T, Chiba N, Kashiwagi K. Systemic arterial stiffness in glaucoma patients. *J Glaucoma*. 2008; 17: 15–18. 18303378 10.1097/IJG.0b013e318098737a

[15] Hulsman CA, Vingerling JR, Hofman A, et al. Blood pressure, arterial stiffness, and open-angle glaucoma: the Rotterdam study. *Arch Ophthalmol*. 2007; 125: 805–812. 17562992 10.1001/archopht.125.6.805

[16] Shim SH, Kim CY, Kim JM, et al. The role of systemic arterial stiffness in open-angle glaucoma with diabetes mellitus. *Biomed Res Int*. 2015; 2015: 425835. 26557669 10.1155/2015/425835 PMC4628752

[17] Turkyilmaz K, Oner V, Cicek Y, et al. Systemic arterial stiffness in patients with pseudoexfoliation glaucoma. *J Glaucoma*. 2014; 23: e108–111. 23632414 10.1097/IJG.0b013e3182955d58

[18] Jia Y, Wei E, Wang X, et al. Optical coherence tomography angiography of optic disc perfusion in glaucoma. *Ophthalmology*. 2014; 121: 1322–1332. 24629312 10.1016/j.ophtha.2014.01.021 PMC4082728

[19] Yarmohammadi A, Zangwill LM, Diniz-Filho A, et al. Peripapillary and macular vessel density in patients with glaucoma and single-hemifield visual field defect. *Ophthalmology*. 2017; 124: 709–719. 28196732 10.1016/j.ophtha.2017.01.004 PMC5499385

[20] Liu L, Jia Y, Takusagawa HL, et al. Optical coherence tomography angiography of the peripapillary retina in glaucoma. *JAMA Ophthalmol*. 2015; 133: 1045–1052. 26203793 10.1001/jamaophthalmol.2015.2225 PMC4950955

[21] Bojikian KD, Chen CL, Wen JC, et al. Optic disc perfusion in primary open angle and normal tension glaucoma eyes using optical coherence tomography-based microangiography. *PLoS One*. 2016; 11: e0154691. 27149261 10.1371/journal.pone.0154691 PMC4858256

[22] Triolo G, Rabiolo A, Shemonski ND, et al. Optical coherence tomography angiography macular and peripapillary vessel perfusion density in healthy subjects, glaucoma suspects, and glaucoma patients. *Invest Ophthalmol Vis Sci*. 2017; 58: 5713–5722. 29114838 10.1167/iovs.17-22865

[23] Ghahari E, Bowd C, Zangwill LM, et al. Association of macular and circumpapillary microvasculature with visual field sensitivity in advanced glaucoma. *Am J Ophthalmol*. 2019; 204: 51–61. 30878489 10.1016/j.ajo.2019.03.004 PMC6642677

[24] Park HY, Shin DY, Jeon SJ, et al. Association between parapapillary choroidal vessel density measured with optical coherence tomography angiography and future visual field progression in patients with glaucoma. *JAMA Ophthalmol*. 2019; 137: 681–688. 30920599 10.1001/jamaophthalmol.2019.0422 PMC6567835

[25] Anderson DR. *Automated Static Perimetry*. St. Louis: The CV Mosby Co.; 1992.

[26] Hodapp E PR, Anderson DR. *Clinical Decisions in Glaucoma*. St Louis: The CV Mosby Co.; 1993.

[27] Yamashina A, Tomiyama H, Arai T, et al. Brachial-ankle pulse wave velocity as a marker of atherosclerotic vascular damage and cardiovascular risk. *Hypertens Res*. 2003; 26: 615–622. 14567500 10.1291/hypres.26.615

[28] Imanishi R, Seto S, Toda G, et al. High brachial-ankle pulse wave velocity is an independent predictor of the presence of coronary artery disease in men. *Hypertens Res*. 2004; 27: 71–78. 15005269 10.1291/hypres.27.71

[29] Whelton PK, Carey RM, Aronow WS, et al. 2017 ACC/AHA/AAPA/ABC/ACPM/AGS/APhA/ASH/ASPC/NMA/PCNA Guideline for the Prevention, Detection, Evaluation, and Management of High Blood Pressure in Adults: Executive Summary: A Report of the American College of Cardiology/American Heart Association Task Force on Clinical Practice Guidelines. *J Am Coll Cardiol*. 2018; 71: 2199–2269.29146533

[30] Fan N, Wang P, Tang L, et al. Ocular blood flow and normal tension glaucoma. *Biomed Res Int*. 2015; 2015: 308505. 26558263 10.1155/2015/308505 PMC4628977

[31] Mozaffarieh M, Flammer J. New insights in the pathogenesis and treatment of normal tension glaucoma. *Curr Opin Pharmacol*. 2013; 13: 43–49. 23092679 10.1016/j.coph.2012.10.001

[32] Buckley C, Hadoke PW, Henry E, et al. Systemic vascular endothelial cell dysfunction in normal pressure glaucoma. *Br J Ophthalmol*. 2002; 86: 227–232. 11815352 10.1136/bjo.86.2.227 PMC1770997

[33] Pache M, Dubler B, Flammer J. Peripheral vasospasm and nocturnal blood pressure dipping–two distinct risk factors for glaucomatous damage? *Eur J Ophthalmol*. 2003; 13: 260–265. 12747647 10.1177/112067210301300304

[34] Cursiefen C, Wisse M, Cursiefen S, et al. Migraine and tension headache in high-pressure and normal-pressure glaucoma. *Am J Ophthalmol*. 2000; 129: 102–104. 10653426 10.1016/s0002-9394(99)00289-5

[35] Drance S, Anderson DR, Schulzer M, et al. Risk factors for progression of visual field abnormalities in normal-tension glaucoma. *Am J Ophthalmol*. 2001; 131: 699–708. 11384564 10.1016/s0002-9394(01)00964-3

[36] Ben-Shlomo Y, Spears M, Boustred C, et al. Aortic pulse wave velocity improves cardiovascular event prediction: an individual participant meta-analysis of prospective observational data from 17,635 subjects. *J Am Coll Cardiol*. 2014; 63: 636–646. 24239664 10.1016/j.jacc.2013.09.063 PMC4401072

[37] Killer HE, Pircher A. Normal tension glaucoma: review of current understanding and mechanisms of the pathogenesis. *Eye (Lond)*. 2018; 32: 924–930. 29456252 10.1038/s41433-018-0042-2 PMC5944657

[38] Mitchell GF. Effects of central arterial aging on the structure and function of the peripheral vasculature: implications for end-organ damage. *J Appl Physiol (1985)*. 2008; 105: 1652–1660. 18772322 10.1152/japplphysiol.90549.2008 PMC2584844

[39] Holwerda SW, Kardon RH, Hashimoto R, et al. Aortic stiffness is associated with changes in retinal arteriole flow pulsatility mediated by local vasodilation in healthy young/middle-age adults. *J Appl Physiol (1985)*. 2020; 129: 84–93. 32437246 10.1152/japplphysiol.00252.2020 PMC7469231

[40] Oettli A, Gugleta K, Kochkorov A, et al. Rigidity of retinal vessel in untreated eyes of normal tension primary open-angle glaucoma patients. *J Glaucoma*. 2011; 20: 303–306. 20577102 10.1097/IJG.0b013e3181e666a1

[41] Hood DC, Raza AS, de Moraes CG, et al. Glaucomatous damage of the macula. *Prog Retin Eye Res*. 2013; 32: 1–21. 22995953 10.1016/j.preteyeres.2012.08.003 PMC3529818

[42] Hwang YH, Jeong YC, Kim HK, et al. Macular ganglion cell analysis for early detection of glaucoma. *Ophthalmology*. 2014; 121: 1508–1515. 24702756 10.1016/j.ophtha.2014.02.019

[43] Cecelja M, Chowienczyk P. Dissociation of aortic pulse wave velocity with risk factors for cardiovascular disease other than hypertension: a systematic review. *Hypertension*. 2009; 54: 1328–1336. 19884567 10.1161/HYPERTENSIONAHA.109.137653

[44] Kim EJ, Park CG, Park JS, et al. Relationship between blood pressure parameters and pulse wave velocity in normotensive and hypertensive subjects: invasive study. *J Hum Hypertens*. 2007; 21: 141–148. 17136108 10.1038/sj.jhh.1002120

[45] Sakuragi S, Abhayaratna WP. Arterial stiffness: methods of measurement, physiologic determinants and prediction of cardiovascular outcomes. *Int J Cardiol*. 2010; 138: 112–118. 19473713 10.1016/j.ijcard.2009.04.027

[46] Spronck B, Heusinkveld MH, Vanmolkot FH, et al. Pressure-dependence of arterial stiffness: potential clinical implications. *J Hypertens*. 2015; 33: 330–338. 25380150 10.1097/HJH.0000000000000407

[47] Rao HL, Pradhan ZS, Weinreb RN, et al. Determinants of peripapillary and macular vessel densities measured by optical coherence tomography angiography in normal eyes. *J Glaucoma*. 2017; 26: 491–497. 28263261 10.1097/IJG.0000000000000655

[48] Muller VC, Storp JJ, Kerschke L, et al. Diurnal variations in flow density measured using optical coherence tomography angiography and the impact of heart rate, mean arterial pressure and intraocular pressure on flow density in primary open-angle glaucoma patients. *Acta Ophthalmol*. 2019; 97: e844–e849. 30900827 10.1111/aos.14089

[49] Shoji T, Zangwill LM, Akagi T, et al. Progressive macula vessel density loss in primary open-angle glaucoma: a longitudinal study. *Am J Ophthalmol*. 2017; 182: 107–117. 28734815 10.1016/j.ajo.2017.07.011 PMC5610624

[50] Prenner SB, Chirinos JA. Arterial stiffness in diabetes mellitus. *Atherosclerosis*. 2015; 238: 370–379. 25558032 10.1016/j.atherosclerosis.2014.12.023

[51] Li CH, Wu JS, Yang YC, et al. Increased arterial stiffness in subjects with impaired glucose tolerance and newly diagnosed diabetes but not isolated impaired fasting glucose. *J Clin Endocrinol Metab*. 2012; 97: E658–E662. 22337914 10.1210/jc.2011-2595

[52] Chung TH, Shim JY, Kwon YJ, et al. High triglyceride to high-density lipoprotein cholesterol ratio and arterial stiffness in postmenopausal Korean women. *J Clin Hypertens (Greenwich)*. 2019; 21: 399–404. 30657241 10.1111/jch.13484 PMC8030508

[53] Fujiwara Y, Chaves P, Takahashi R, et al. Relationships between brachial-ankle pulse wave velocity and conventional atherosclerotic risk factors in community-dwelling people. *Prev Med*. 2004; 39: 1135–1142. 15539047 10.1016/j.ypmed.2004.04.026

[54] Tomiyama H, Hashimoto H, Tanaka H, et al. Continuous smoking and progression of arterial stiffening: a prospective study. *J Am Coll Cardiol*. 2010; 55: 1979–1987. 20430271 10.1016/j.jacc.2009.12.042

[55] Tomiyama H, Shiina K, Vlachopoulos C, et al. Involvement of arterial stiffness and inflammation in hyperuricemia-related development of hypertension. *Hypertension*. 2018; 72: 739–745. 29987103 10.1161/HYPERTENSIONAHA.118.11390

[56] Tang B, Luo F, Zhao J, et al. Relationship between body mass index and arterial stiffness in a health assessment Chinese population. *Medicine (Baltimore)*. 2020; 99: e18793. 32011479 10.1097/MD.0000000000018793 PMC7220472

[57] Lioufas NM, Pedagogos E, Hawley CM, et al. Aortic calcification and arterial stiffness burden in a chronic kidney disease cohort with high cardiovascular risk: baseline characteristics of the impact of phosphate reduction on vascular end-points in chronic kidney disease trial. *Am J Nephrol*. 2020; 51: 201–215. 32023606 10.1159/000505717

[58] Yoshida M, Tomiyama H, Yamada J, et al. Relationships among renal function loss within the normal to mildly impaired range, arterial stiffness, inflammation, and oxidative stress. *Clin J Am Soc Nephrol*. 2007; 2: 1118–1124. 17913969 10.2215/CJN.01880507

